# State reliability analysis methods for nonlinear systems integrating adaptive entropy weighted grey relation and Dempster-Shafer’s Theory

**DOI:** 10.1371/journal.pone.0340886

**Published:** 2026-02-13

**Authors:** Liming Gou, Jian Zhang, Lin Qi, Lihao Wen

**Affiliations:** 1 College of Management Science and Engineering, Beijing Information Science & Technology University, Beijing, China; 2 Laboratory of Big Data Decision making for Green Development, Beijing, China; 3 Beijing International Science and Technology Cooperation Base of Intelligent Decision and Big Data Application, Beijing, China; 4 Beijing Knowledge Management Research Center, Beijing, China; 5 School of Electronic and Information Engineering, University of Science and Technology Liaoning, Liaoning, China; The University of British Columbia, AUSTRALIA

## Abstract

Under uncertain environmental conditions, a nonlinear system may encounter problems like information conflicts, ambiguity, loss, and unclear interdependencies, which can result in low accuracy in predicting abnormal system states. System failures lead to negative impacts. To address these issues, this study proposes an analysis model that incorporates factor weight adaptive adjustment and integrates Dempster-Shafer’s theory (D-S theory) algorithms to construct an evaluation model, and to quantify uncertainty factors and correlation factors within the system. This approach reduces the impact of uncertainty in information features on analysis accuracy, enhances the precision of system reliability state probability assessment, and improves decision-making management levels. The results of the wind turbine system case study indicate that the proposed algorithm achieves a system state identification accuracy of 97% and a system reliability probability of 65%, with an overall accuracy improvement of 4.5% compared to traditional algorithms and a reliability probability assessment accuracy improvement of 5.22%, better aligning with the actual system’s state probability distribution.

## Introduction

System state reliability is a critical factor influencing the efficient operation of a system. Since the early 20th century, scholars have dedicated significant effort to researching system reliability. From the perspective of technological development, system reliability assessment can be summarized into three phases: the nascent stage of reliability development and the initial phase based on extensive reliability testing, the stage of sensor technology advancement, and the stage of intelligent development. With continuous technological advancement, system reliability research has expanded from initial component parameter design, reliability testing implementation, and reliability assessment metrics development, to achieving real-time monitoring of system state reliability, achieving significant progress [1]. The complexity of system structures and operational environments leads to the diversification of system tasks, nonlinear coupling with components, and temporal changes of system states. The efficient operation of systems is impacted by the introduction of complex interdependent influence relationships by these factors [2].

Since the 1980s, advancements in sensor technology and its applications have enhanced the precision of system state information monitoring. Some scholars observed uncertainty and conflicting factors in system state information [3–4], which lead to mismatches in system reliability assessments. The development of system reliability assessment theories based on probability and statistics to preliminarily quantify information uncertainty and conflict issues was prompted by this phenomenon, which drew scholarly attention [5–6]. These probabilistic and statistical reliability assessment theories initially addressed uncertainty and conflict within state information, which enabled analysis of system state reliability. However, as systems become increasingly intelligent, state information exhibits characteristics such as large volume and multimodality, persisting inaccuracies in reliability assessment. This raises the question: Are existing methods for quantifying information uncertainty and conflict sufficient to support the current demands of assessing the reliability of multimodal, large-volume systems?

During the current stage of nonlinear system state reliability assessment, information remains subject to uncertainties such as fuzziness, gray areas, and randomness, which makes it difficult to precisely resolve the mismatch between information feature representation and state reliability correspondence, making the analysis of system state reliability increasingly important. The uncertainty in state information significantly affects the extraction and analysis of information features. Many scholars have studied the reliability of nonlinear systems by focusing on the uncertainty in state information. While some algorithms have addressed prior probability issues, uncertainties regarding inter-information correlations persist, leaving room for improvement in the precision of system state reliability assessment.

Building upon prior research, this study analyzes the impact of uncertainty in the interrelationships among system state information on its reliability assessment. Establishes a framework for system state reliability assessment, investigates an analysis model based on factor weight adaptive adjustment, and explores an entropy-weighted grey correlation system reliability assessment algorithm based on interval characteristic quantities. By introducing a weight-adaptive adjustment mechanism, it improves the integrated analysis of multi-factor indicators. This approach quantifies uncertainty factors and interrelated elements within the system, thereby mitigating the impact of information uncertainty on the precision of system state reliability assessment. This provides a theoretical foundation for system state reliability evaluation and offers supportive, reference-worthy guidance for operational management at the decision-making level.

## Related work

From a technological perspective, research on the state reliability of nonlinear systems can be divided into three stages: the stage based on extensive reliability testing, the sensor monitoring technology stage, and the intelligent stage. Deductive analysis, statistical inference, and data-driven modeling are the main methods of reliability analysis for nonlinear systems from a methodological perspective. Forward reasoning is used in deductive analysis methods to analyze system states. While statistical inference methods are based on statistics, they employ statistical analysis of state information to assess the reliability of the system. Data-driven modeling utilizes big data analysis techniques to predict system reliability states.

Complex system structures, information uncertainty, and scarcity of effective information are frequently observed in the reliability analysis of nonlinear system states. Grey system theory demonstrates broad applicability in decision-making processes involving systems with uncertain information, providing an optimal approach for multi-factor decision problems [7]. Deng Julong proposed the GRA theory in 1982 as one of the branches of grey system theory. It quantifies the correlation between features by analyzing the geometric curve trends of different information features, with relatively low requirements for the regularity of information [8]. This theory demonstrates excellent applicability to information systems containing uncertainty. It is now widely applied in fields such as industry, agriculture, environment, economy, society, management, transportation, and petroleum [9–13].

To address the impact of information uncertainty on the system state reliability analysis. Cheng et al. attempted to use the GRA method to analyze the interrelationships among state data of numerically controlled machine tools, combined with the analytic hierarchy process (AHP) to obtain state information weights, thereby resolving the reliability allocation issue for machine tool systems [14]. Wu et al combined the grey-TOPIS method and the weighted rank sum method to assess the risk of water inrush from coal seam floors, providing a new method for deep coal seam mining [15]. Zhang et al addressed the issue of fuzzy risk in liquefied natural gas loading and unloading operations by using the GRA method for human reliability analysis [16]. It identified fuzzy rules and estimated human error rates through weighting rules, and validated its findings through actual engineering cases. Ghoushchi et al proposed a method combining the GRA method and SWARA to assess the weights of risk factors for solar panel failures, determining failure priorities through failure priority ranking [17]. Hua et al integrated GRA with failure mode and effects analysis (FMEA) to achieve more accurate risk ranking [18]. GRA plays a significant role when dealing with uncertain influencing factors.

The effectiveness of selecting and estimating risk indicators in nonlinear systems with multiple coexisting indicators has a significant impact on the final analysis results. Information entropy is a measure of the degree of uncertainty in information [19]. As the information entropy increases, the distribution of information states becomes more chaotic. Therefore, information entropy can be used to determine the value of a particular piece of information to the occurrence of an event, thereby adjusting the weight of that information feature [20]. Approximate entropy, fuzzy entropy, distance entropy, and cross-entropy have been developed in management science, computer science, and artificial intelligence to solve state monitoring and system decision-making domains [21–25]. Dehkordi et al extracted significant risk indicators for water resource management under drought conditions by combining fuzzy entropy with the fuzzy additive ratio assessment (ARAS) model in their indicator weight analysis [26]. The reliability of the algorithm was improved by incorporating entropy, as indicated by the results. Yang et al proposed an improved entropy weighting method combined with fuzzy comprehensive evaluation for assessing complex environmental systems, which mitigates excessive correction of normal data in weight determination [27]. Xia et al. addressed the challenge of information feature extraction by proposing a fault diagnosis framework that combines the computational stability advantages of hierarchical refined composite multi-scale fuzzy entropy (HRCMFE) and hierarchical entropy (HE), using an adaptability function for evaluation, thereby improving the accuracy of information feature extraction and system fault identification [28].

The reliability analysis of the system may be affected by conflicts between information features due to information uncertainty. D-S theory is an extension of probability theory that can better handle information conflict issues [29]. Tang et al. argued that traditional D-S theory struggles with evidence conflicts in Failure Mode and Effects Analysis (FMEA). By integrating fuzziness metrics with FMEA, they enhanced the efficiency and precision of D-S theory in risk assessment [30]. To solve the problem of intuitive contradictions caused by evidence conflicts in fusion, Xiao devised a D-S theory fusion algorithm that utilizes evidence belief divergence and trust entropy [31]. The accuracy of system operational state analysis can be improved by using the Jensen-Shannon divergence to measure the differences and conflicts between evidence. In response to the issues of evidence conflicts and uncertainty, Tang et al proposed a new reliability coefficient based on the D-S theory, incorporating the commitment evidence distance, which effectively addressed the impact of information conflicts and uncertainty on information fusion [32].

In summary, entropy to some extent highlights the intrinsic information preferences of a system, while D-S theory mitigates the interference of conflict factors based on the objectivity of information [33]. D-S theory algorithms are still unable to quantify conflict and uncertainty metrics due to the uncertainty of information. Therefore, this paper combines GRA, entropy, and D-S theory based on D-S theory research to measure the degree of association and uncertainty between information, to improve the precision of quantifying information uncertainty, and to make the reliability analysis of nonlinear system states more accurate.

## Materials and methods

### D-S theory

As an effective reasoning algorithm tool for expressing uncertainty information, the D-S theory [34] addresses the shortcomings of Bayesian algorithms that determine posterior distributions and classifications based on prior distributions [35], without imposing stringent requirements on prior data. It is currently widely applied in the fields of system state reliability assessment, intelligent analysis, and decision-making.

D-S theory assumes a non-empty finite set *Θ={A*_*1*_*, A*_*2*_*,…, A*_*n*_*}* for a given problem, where the *n* elements are mutually incompatible. This set *Θ* is termed an identification framework. *A*_*i*_ is a subset of *Θ*, where *i*∈[*1,n*]. *2*^*Θ*^ is the power set of *Θ*. The fundamental distribution function is a mapping from the power set to [0,1], denoted as a mass function and represented by *m*. This function satisfies the conditions of Eq. (1).


m(ϕ)=0;∑m(A)=1 (1)

Where *φ* denotes an impossible event, *m* is termed the basic probability allocation function (BPA) on the identification framework *Θ* [36], with *m(A)* representing the degree of support evidence provides for the described event. If *m(A) > 0*, then *A* is referred to as a focal element of the function.

Confidence represents the degree to which a data feature supports an event outcome, the reliability of evidence information. The confidence function within the recognition framework can be expressed as Eq. (2).


$$Bel\left(A\right)=\sum_{B\subseteq A}m\left(B\right)$$ (2)

This formula expresses that the credibility function of an event is the sum of the support probabilities of all its subsets.

Based on the above analysis, for basic evidence fusion, assume two pieces of evidence *E*_*1*_ and *E*_*2*_ under the same recognition framework *Θ*, with credibility functions *m*_*1*_ and *m*_*2,*_ respectively, and focal points *A*_*1*_ and *A*_*2,*_ respectively. The fusion rule is shown in Eq. (3).


$$E_{1}⊕E_{2}=\frac{\text{1}}{1-K}\sum A_{1}\cap A_{2}=Am_{1}\left(A_{1}\right)m2\left(A_{2}\right)$$ (3)

Among these, $K=\sum_{A_{1}\cap A_{2}=\phi }m_{1}\left(A_{1}\right)m2\left(A_{2}\right)$ is the evidence conflict coefficient, and $\frac{\text{1}}{1-K}$ is the normalized coefficient after conflict removal.

Thus, when there are *n* pieces of evidence, the fusion rule is as shown in Eq. (4).


$$E_{1}⊕E_{2}...⊕E_{n}=\frac{\text{1}}{1-K}\sum A_{1}\cap ...\cap A_{n}=Am_{1}\left(A_{1}\right)m2\left(A_{2}\right)...m_{n}\left(A_{n}\right)$$ (4)

In which $K=\sum_{A_{1}\cap ...\cap A_{n}=\phi }m_{1}\left(A_{1}\right)m2\left(A_{2}\right)...m_{n}\left(A_{n}\right).$

### Grey relation analysis

Uncertainty and ambiguity exist in system reliability analysis [37]. When the changing trends of two types of indicator information in system reliability analysis converge, the indicators exhibit high correlation. When the changing trends of indicator information differ significantly, uncertainty conflicts arise between the indicators. GRA primarily achieves the analysis of system state changes through steps such as correlation coefficient calculation, correlation degree calculation, and statistical analysis, and selects an information set that reflects the reliability of the system state [38].

Assume that the original information feature set is *x=(x*_*i*_*(1), x*_*i*_*(2),…, x*_*i*_*(n))*. The analysis process is facilitated by the normalization of different types of information features to eliminate the interference of dimensions. The normalization calculation can be seen in Eq. (5).


$$x_{i}\left(k\right)=x_{i}\left(k\right)/x_{0}$$ (5)

Where, *k = 1, 2, …, n*; *x*_*0*_ > *0.* In addition, the information is analyzed and compared, and the correlation coefficient between them is calculated, as shown in Eq. 6).


$$\begin{array}{c} {}*20cr_{ij}^{+}=\frac{\underset{i}{\min}\underset{j}{\min}\left|x_{ij}-X^{+}\right|+\xi \underset{i}{\max}\underset{j}{\max}\left|x_{ij}-X^{+}\right|}{\left|x_{ij}-X^{+}\right|+\xi \underset{i}{\max}\underset{j}{\max}\left|x_{ij}-X^{+}\right|}\\ r_{ij}^{-}=\frac{\underset{i}{\min}\underset{j}{\min}\left|x_{ij}-X^{-}\right|+\xi \underset{i}{\max}\underset{j}{\max}\left|x_{ij}-X^{-}\right|}{\left|x_{ij}-X^{-}\right|+\xi \underset{i}{\max}\underset{j}{\max}\left|x_{ij}-X^{-}\right|} \end{array}$$ (6)

Among them, X+=max1≤i≤m1≤j≤nxij={x1+,x2,+⋯,xn+}, X−=min1≤i≤m1≤j≤nxij={x1−,x2,−⋯,xn−}, represent the forward optimal sequence and the backward worst sequence, respectively, $\underset{i}{\min}\underset{j}{\min}$, $\underset{i}{\max}\underset{j}{\max}$ represent the maximum and minimum differences of the forward or backward sequence of the matrix set, ξ represents the resolution coefficient, ξ∈[0,1], and it is generally set to 0.5. Based on this, the comprehensive grey correlation coefficient *r*_*ij*_ can be expressed by Eq. (7).


rij=(1+rij+/1+rij+rij−\nulldelimiterspacerij−)−2 (7)

In real analysis, there are multiple indicators, and each plays a different role in the analysis. Therefore, the gray correlation degree *r*_*u*_*(I)* can be expressed as Eq. (8).


$$r_{u}\left(I\;\right)=\frac{\text{1}}{m}{\left|\sum_{i=1}^{m}{\left(r_{ij}\right)}^{q}\right|}^{\frac{\text{1}}{q}}$$ (8)

In general, *q* is set to 2.

### Entropy-based weighting of information indicators

Due to the complexity of the original information set, such as similarities, contradictions, missing data, and anomalies, the validity of Eq. (8) is reduced, which affects the accuracy of the algorithm and makes it unable to meet certain requirements in the current analysis. The measure of uncertainty in information is known as entropy. Adjusting the weights of various influencing factors using entropy can improve both the effectiveness of information extraction and the identification of information uncertainty [39]. By integrating entropy weights with GRA, the weights under factor associations can be redistributed from the perspective of effective information content and information relationality, effectively reducing algorithmic errors and improving algorithmic precision.

Entropy is connected to the flow of information. Different time points exhibit differences in the fluctuations and trends of parameter characteristics. The uncertainty function of information flow transmission is related to the amount of uncertainty in the information, the weights of different factors, and the warning threshold, as shown in Eq. (9).


f(t)→(u(x),w,θ) (9)

Information flows through the entire system analysis. The logarithmic sequence can be processed by assigning weights, which identify the usability of the information flow and the effective components within it. Forward approximation and backward approximation differ in processing the logarithmic sequence when multiple factors have different weights. The dataset can be standardized by adopting forward and backward approximation concepts. Eq. (10) displays the transformed calculation formula.


$${x_{ij}}^{+}=\frac{x_{ij}-\min x_{i}}{\max x_{i}-\min x_{i}},\;\;\;\;{x_{ij}}^{-}=\frac{\max x_{i}-x_{ij}}{\max x_{i}-\min x_{i}}$$ (10)

Where *i* represents the analysis object, and *j* represents the influencing factor.

Let $y_{ij}=\{{x_{ij}}^{+},\;\;{x_{ij}}^{-}\}$, then under the condition of influencing factor *j*, the weight of the system under target *i* can be expressed by Eq. (11).


$$Y={\left(Y_{ij}\right)}_{m\times n}=y_{ij}{\left[\sum_{i=1}^{m}y_{ij}\right]}^{-1}$$ (11)

Calculate the entropy value of the influencing factor *j*, and its entropy value can be expressed by Eq. (12).


$$S_{j}=-\sum_{i=1}^{m}\left(Y_{ij}\ln Y_{ij}\right)/\ln n$$ (12)

Then, the entropy weight of the influencing factor *j* can be expressed by Eq. (13).


$$\omega_{j}=\left(1-S_{j}\right){\left[n-\sum_{j=1}^{n}S_{j}\right]}^{-1}$$ (13)

Where, *w*_*j*_∈*[0,1]*, *j=1, 2, …, n*.

Multiple factors influence the uncertainty function of information. Its equation related to uncertainty can be expressed by Eq. (14).


U(x)=f(u(x),e) (14)

Where *u(x)* is the uncertainty function and *e* is the influencing factor.

Therefore, the uncertainty of information can be expressed as *U(x*_*1*_*)*, *U(x*_*2*_*)*, …, *U(x*_*n*_*)*. Thus, the uncertainty after fusion of information features can be expressed by Eq. (15).


$$U\left(E_{1}⊕E_{2}⊕...⊕E_{n}\right)=f\left(U\left(x_{1}\right),\;U\left(x_{2}\right),\;\ldots ,U\left(x_{n}\right)\right)$$ (15)

The degree of information flow transmission causes uncertainty. If the uncertainty of information is somehow related to the probability of risk occurrence, then it can be considered that the uncertainty of information follows an exponential distribution to a certain extent, as shown in Eq. (16).


$$u\left(t\right)=\lambda {e^{-}}^{\lambda t}$$ (16)

The probability of uncertainty occurring under abnormal conditions in the reliability of the system state can be expressed by Eq. (17).


$$U\left(t\right)=\int_{0}^{t}\lambda {e^{-}}^{\lambda t}dt$$ (17)

By combining entropy weights with GRA, information can be quantified from the perspectives of correlation and uncertainty, and effective information can be extracted. This can be applied to reliability analysis algorithms, thereby improving the accuracy of the analysis.

## Algorithm improvements

### Analysis of information fusion framework

**Fig 1:**
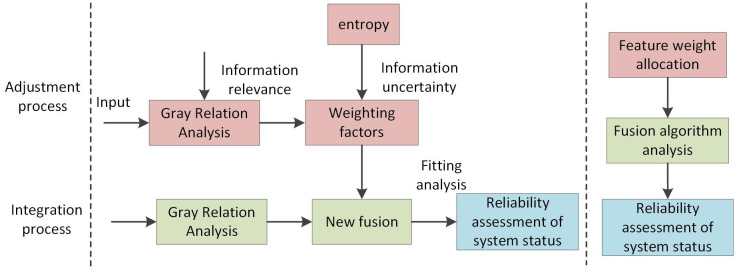
The integration diagram of gray correlations with entropy-weighted correlations.

Divide the process of analyzing the reliability of the system state into two stages: the adjustment process and the fusion process. Adjusting the weights of factors that influence system operational reliability is the main part of the adjustment process. This is achieved by analyzing the relationships between various system components and analyzing information from different sources to clarify the interrelationships between factors, thus adjusting the weights of multi-state, multi-factor variables. The integration process primarily involves using integration algorithms to conduct integrated analysis of multi-factor indicators, providing a theoretical basis for system state reliability assessment and offering reference-based assistance for operational decision-making at the decision-making level. The diagram of the integration of gray correlations with entropy is shown in Fig 1.

During the adjustment process, GRA is combined with entropy to analyze the interrelationships and validity of information, discarding features with high variability. The accuracy of the fusion algorithm from multiple dimensions is improved by redistributing information weights. During the fusion process, GRA is combined with D-S theory to derive a new fusion algorithm. The analysis set and reference set are analyzed to determine the uncertainty of the system’s current state.

**Fig 2:**
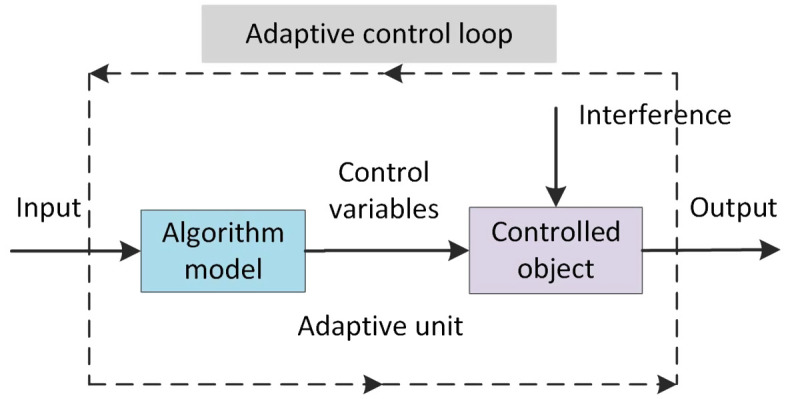
Weight adaptive adjustment diagram.

During system operation, the collected information changes dynamically over time, and the analysis of information uncertainty varies depending on the period and state. [40]. Dynamic adaptive regulation is present in the factor weights obtained using the entropy weight method, which can be referred to as a typical nonlinear time-varying system. The controlled object in this system is the factors that relate to the system state, and the control variable is the weight of the factors. An adaptive unit is formed by the algorithm model, controlled object, and control variable. Fig 2 illustrates certain interference factors in the regulation loop.

The identification of information uncertainty during the adaptive weight adjustment process leads to the measurement of information importance. The fusion of system state reliability reduces the impact of uncertainty on fusion analysis, resulting in improved fusion accuracy.

### Analysis of algorithm improvements

The process of determining information weights can be unstable and inaccurate due to the complexity of information, which includes disorder, loss, redundancy, and anomalies. Therefore, it is necessary to identify and extract interval features from the original data, eliminate factors and features of lower value, and, based on the interval feature vector, conduct research on system state reliability analysis algorithms from the perspective of feature fusion.

Based on the standardized processing of positive and negative data sets, the positive and negative entropy of the data sets are calculated separately, as shown in Eq. (18).


$$S_{j}^{+}=(-\sum_{i=1}^{m}\left(Y_{ij}^{+}\ln Y_{ij}^{+}\right)/\ln n);\;\;\;S_{j}^{-}=(-\sum_{i=1}^{m}\left(Y_{ij}^{-}\ln Y_{ij}^{-}\right)/\ln n)$$ (18)

The degree to which a factor supports an event is determined by its weight. It calculates factor weights separately from positive and negative directions, and then weights the weights obtained from positive standardization and negative standardization to balance the impact of it on the extraction of information validity, thereby resulting in factor weights, as shown in Eq. (19).


$$\omega_{j}=\omega_{j}^{+}+\omega_{j}^{-}=\left(1-S_{j}^{+}\right){\left[n-\sum_{j=1}^{n}S_{j}^{+}\right]}^{-1}+\left(1-S_{j}^{-}\right){\left[n-\sum_{j=1}^{n}S_{j}^{-}\right]}^{-1}$$ (19)

A matrix of entropy weights under each objective is obtained after the calculation. The obtained entropy weights are normalized, as shown in Eq. (20).


$$\omega j\prime =\omega_{j}/\sum_{i=1}^{n}\omega j$$ (20)

The weighted membership degree formula for the features is the product of a factor’s entropy weight and its weight under different evaluation objects, as shown in Eq. (21).


$$X={\left[x_{ij}\right]}_{m\times n}={\left[\omega j^{\prime }*Y_{ij}\right]}_{m\times n}$$ (21)

Information value is reflected in features. The more valuable features that are extracted during the analysis of a system’s reliability, the more advantageous it will be for the analysis results. The objective’s support and negation are both valuable. The weighted membership degree of factors is calculated using features, as demonstrated in Eq. (22).


$$<X>={\left[<x_{ij}>\right]}_{m\times n}={\left[<\omega_{j}>*<Y_{ij}>\right]}_{m\times n}$$ (22)

Here, *< X>* represents the feature values of the dataset X.

By calculating the maximum and minimum values of the weighted membership degrees of the factor features, it obtains the maximum and minimum extreme differences of the new matrix set in the positive or negative direction $\underset{i}{\max}\underset{j}{\max}$, $\underset{i}{\min}\underset{j}{\min}$ $r_{ij}^{+}$ and $r_{ij}^{-}$ are functions related to extreme differences and will change accordingly, as shown in Eq. (23).


$$\begin{array}{c} {}*20cr_{ij}^{+}=\frac{\underset{i}{\min}\underset{j}{\min}\left|<x_{ij}>-<X^{+}>\right|+\xi \underset{i}{\max}\underset{j}{\max}\left|<x_{ij}>-<X^{+}>\right|}{\left|<x_{ij}>-<X^{+}>\right|+\xi \underset{i}{\max}\underset{j}{\max}\left|<x_{ij}>-<X^{+}>\right|}\\ r_{ij}^{-}=\frac{\underset{i}{\min}\underset{j}{\min}\left|<x_{ij}>-<X^{-}>\right|+\xi \underset{i}{\max}\underset{j}{\max}\left|<x_{ij}>-<X^{-}>\right|}{\left|<x_{ij}>-<X^{-}>\right|+\xi \underset{i}{\max}\underset{j}{\max}\left|<x_{ij}>-<X^{-}>\right|} \end{array}$$ (23)

Therefore, by comprehensively considering the interrelationships among factors and the degree of uncertainty in the information, and after eliminating the uncertain factors in the information, the evidence of the fusion function under the influence of multiple factors can be expressed as Eq. (24).


$$m_{j}\left(i\right)=\left[1-r_{u}\left(I\;\right)\right]Y_{ij}$$ (24)

In which the gray correlation degree *r*_*u*_*(I)* is a function of the correlation coefficients $r_{ij}^{+}$ and $r_{ij}^{-}$ between pieces of information, and changes with their changes.

GRA theory has the ability to effectively measure uncertainty and fuzzy logical relationships within a system. By integrating the entropy-weighted gray relational algorithm with the evidence theory algorithm, the new *mass* function exhibits cases where the sum does not equal 1. The two factors that affect this phenomenon are the trend of data changes, specifically the magnitude of the extracted feature values, and the removal of uncertainty factors obtained through entropy-weighted gray relational calculations. When calculating the credibility function, normalization processing is necessary.

An improved algorithm for the fusion of entropy-weighted gray correlation based on interval feature values is proposed in this section. By incorporating interval feature values into the quantification analysis of association and uncertainty, this approach avoids inaccuracies in the calculation of indicator weight factors caused by data complexity and errors resulting from human subjective factors. It simplifies the model for analyzing system state reliability, effectively preserves the valid information in the original data, reduces the interference and impact of factors such as data complexity and uncertainty on the analysis process, and enhances the accuracy of model calculations.

## Experiment results and analysis

### Experimental Steps

Based on the algorithm proposed in this paper, the experimental steps are outlined as follows:

(1) Screen samples and calculate their feature values.
(2) Standardize sample feature values and calculate their weights.
(3) Calculate the weighted entropy of sample feature values to obtain the entropy weight function, then compute adaptive weighted membership degrees after entropy is introduced.
(4) Calculate the grey correlation coefficients between features and uncertainty confidence under the correlation function.
(5) Perform D-S fusion calculation on feature evidence values using correlation coefficients.
(6) Calculate confidence and uncertainty probability under the fusion of entropy-weighted grey correlation and D-S theory.
(7) Compare accuracy before and after algorithm improvement.

### Uncertainty knowledge analysis of the wind turbine system

System state reliability refers to the ability of a system to consistently perform its fundamental tasks over a given period of time. The overall reliability of the system’s status is determined by the reliability of each component in the system. Eq. (25) displays the overall and component relationships of the system’s status.


$$S_{i}\left(t\right)=\left\{S1\left(t\right),S2\left(t\right),S3\left(t\right),\ldots ,Sn\left(t\right)\right\}$$ (25)

Where *S*_*i*_*(t)* represents the status of a component in the system at time *t*.

The primary purpose of the analysis of system states is to identify system states and potential issues [41]. The manifestation of system state reliability differs due to the complex series and parallel relationships among system components. Different components exhibit inconsistencies in the state characteristics they exhibit in different states. Therefore, the system state *S(t)* can be expressed as shown in Eq. (26).


$$S\left(t\right)=\cup S_{i}\left(t\right)$$ (26)

Analysis of the reliability probability of a modular wind turbine system. The probability of each component’s reliability in a certain state is related to the probability of it occurring in a certain state risk, which can be specifically expressed by Eq. (27).


$$P\left(t\right)=\left[\begin{array}{c} P_{11}\left(t\right),P12\left(t\right),P13\left(t\right),P14\left(t\right)\\ P_{21}\left(t\right),P22\left(t\right),P23\left(t\right),P24\left(t\right)\\ ...\\ P_{n1}\left(t\right)P2\left(t\right),P3\left(t\right),P4\left(t\right) \end{array}\right]$$ (27)

Where *P*_*ij*_*(t)* represents the reliability probability of component *i* being in state *j*, with *i ≤ n* and *j = 1,2,3,4*.

### Calculation of status information characteristics for wind turbine systems

The experimental data is sourced from the New Energy Research Institute of China Electric Power Research Institute. Collected at a wind farm in Liaoning Province, China, these data are publicly available for scientific research purposes without requiring additional permission.

By conducting a statistical analysis of the cumulative abnormal information occurring throughout the year for wind turbines at the wind farm, it was found that the probability of abnormal conditions occurring in components such as generators, gearboxes, shafts, and pitch systems was relatively high. The probability of abnormal conditions occurring in two different wind turbine models was similar, as determined by an analysis of the probability. The probability of abnormal conditions occurring in the same components was generally similar based on the analysis of individual turbines and turbine groups. This indicates that environmental and other external factors that influence the reliability of the turbine system are reflected in the system’s reliability information. The use of turbine status data as a starting point for system reliability research is valuable.

Considering that the gearbox is one of the critical components of the wind turbine system and also a part with a relatively high incidence of abnormal conditions, its normal operation directly impacts the overall state reliability and operational efficiency of the wind turbine. Therefore, taking the wind turbine gearbox as the research object, it assesses the reliability of the system under conditions where only gearbox alarm information is present for a certain period.

Analyze the status of the wind turbine system. Continuous state phenomena are the predominant characteristic of the operational status of wind turbine systems, with distinct features of data continuity. The analysis of discrete data information volume involves the use of information entropy, which serves as a measure of information uncertainty. To obtain discrete data, the gearbox status information is sampled at points under time series patterns. Based on the system’s reliability performance over the past year, risk data codes are classified. System states are categorized into four modes according to their impact severity on wind turbine reliability: normal states *F*_*0*_, latent risk states *F*_*1*_, explicit risk states *F*_*2*_, and alarm risk states *F*_*3*_. This classification is specifically expressed by Eq. (28).


State={F0,F1,F2,F3} (28)

In a latent risk environment, interference with the system is minimal. In an explicit risk environment, the system can operate but will experience a significant impact on certain performance aspects, thereby disrupting overall system efficiency, accuracy, and stability. Alarm risk represents a more severe hazard, typically those capable of causing significant system disruption or downtime. Different state modes indicate varying reliability conditions. State data within each mode is interval-based, categorized according to risk severity-mapped data ranges. All metric feature results are calculated as the average of the interval means.

Through knowledge analysis of the features, it was found that the information related to gearbox state reliability primarily includes gearbox oil pressure, high-speed bearing temperature, low-speed bearing temperature, inlet oil temperature, gearbox oil temperature, and rotor speed. A discrete dataset is formed by collecting data every 5 minutes, with all experimentaldata representing instantaneous averages.

Table 1 displays the basic dataset used in the experiment.

**Table 1:** Base dataset.

*Time*	*Gearbox high-speed bearing temperature*	*Gearbox low-speed bearing temperature*	*Gearbox oil pressure*	*Gearbox inlet oil temperature*	*Gearbox oil temperature*	*Wind turbine rotation speed*
1	70	105	1	26	91	5
2	183	155	76	19	120	98
3	360	257	75	9	158	98
4	455	308	74	11	170	98
5	512	335	74	15	172	98
6	545	352	73	21	176	98
7	565	363	72	29	182	98
8	579	372	70	39	189	98
9	588	379	68	52	198	100
10	600	387	68	46	209	109
…	…	…	…	…	…	…
…	…	…	…	…	…	…
…	…	…	…	…	…	…
n	535	484	44	330	440	98

The initial organization, screening, and analysis of data related to gearboxes. The rotor speed is a reflection of the force exerted on the turbine due to wind direction deviation, and wind speed is one of the factors that has a specific impact on wind turbines. The system may be in different states due to inconsistent changes in rotor speed, and additional analysis based on other relevant data is necessary. Excluding cases where the wind turbine does not operate due to no wind, 200 representative sample intervals were selected under wind speed conditions, including 134 normal state samples, 62 latent risk state samples, 2 explicit risk state samples, and 2 alarm state samples.

The mean, variance, root mean square, kurtosis, and other representative parameter values of the data for the gearbox high-speed bearing temperature, gearbox low-speed bearing temperature, gearbox oil pressure, gearbox inlet oil temperature, and gearbox oil temperature in the sample intervals were calculated to gain an initial understanding of the trends in data characteristics. These were then labeled in order as *E*_*1*_, *E*_*2*_, *E*_*3*_, *E*_*4*_, *E*_*5*_, as shown in Table 2.

**Table 2:** Basic feature parameters of the gearbox dataset.

Evidence	$\overset{―}{X}$	$\sigma_{x}^{2}$	*X*_*rms*_	*K*_*u*_
*E*_*1*_	0.0644	0.0981	1.9674	0.0017
*E*_*2*_	0.0528	0.0763	1.7098	0.0004
*E*_*3*_	0.1452	0.1693	3.0764	0.0001
*E*_*4*_	0.1635	0.2191	3.4846	0.0001
*E*_*5*_	0.0522	0.0816	1.7534	0.0005

Using a traditional evidence-fusion algorithm on the selected samples, the number of intervals identified for the system in different states was 145, 53, 1, and 1, respectively. There were unequal amounts of data in each interval, with an overall identification accuracy of 92.5%.

The analysis process is affected by uncertainty throughout due to the uncertainty of information, which affects the reliability assessment of the system state and the recognition accuracy of the algorithm. Therefore, when performing fusion analysis on the feature values, it is also necessary to use relevant algorithms to quantify the uncertainty introduced by the information, thereby mitigating the impact of uncertainty on the accuracy of the model algorithm.

### Reliability analysis of wind turbine systems considering uncertainty

The characteristics of a wind turbine are subject to dynamic changes. Representative sample interval characteristic values selected through screening are used for algorithm application analysis.

First, the characteristic value data set is standardized to calculate $x_{ij}^{+}$ and $x_{ij}^{-}$.


$$x_{ij}^{+}=\left[\begin{array}{ccccc} {}*20c0.0319 & 0.0307 & 0.0472 & 0.0469 & 0.0295\\ 0.0490 & 0.0444 & 0.0550 & 0.0628 & 0.0463\\ 1.0000 & 1.0000 & 1.0000 & 1.0000 & 1.0000\\ 0.0000 & 0.0000 & 0.0000 & 0.0000 & 0.0000 \end{array}\right],$$

$$x_{ij}^{-}=\left[\begin{array}{ccccc} {}*20c0.9681 & 0.9693 & 0.9528 & 0.9531 & 0.9705\\ 0.9510 & 0.9556 & 0.9450 & 0.9372 & 0.9537\\ 0.0000 & 0.0000 & 0.0000 & 0.0000 & 0.0000\\ 1.0000 & 1.0000 & 1.0000 & 1.0000 & 1.0000 \end{array}\right].$$

Under the influence of factor *j*, the weights of the system under target *i* are calculated separately for the positive and negative directions.


$$Y^{+}=\left[\begin{array}{ccccc} {}*20c0.0295 & 0.0285 & 0.0428 & 0.0423 & 0.0274\\ 0.0454 & 0.0413 & 0.0499 & 0.0566 & 0.0430\\ 0.9251 & 0.9302 & 0.9073 & 0.9011 & 0.9296\\ 0.0000 & 0.0000 & 0.0000 & 0.0000 & 0.0000 \end{array}\right],$$

$$Y^{-}=\left[\begin{array}{ccccc} {}*20c0.3316 & 0.3314 & 0.3288 & 0.3298 & 0.3319\\ 0.3258 & 0.3267 & 0.3261 & 0.3242 & 0.3261\\ 0.0000 & 0.0000 & 0.0000 & 0.0000 & 0.0000\\ 0.3426 & 0.3419 & 0.3451 & 0.3460 & 0.3420 \end{array}\right].$$

Analyze the entropy values of the characteristics under various influencing factors.


$$S_{j}^{+}=\left[\;\begin{array}{ccccc} {}*20c0.2282 & 0.2167 & 0.2689 & 0.2814 & 0.2177 \end{array}\;\right],$$

$$S_{j}^{-}=\left[\begin{array}{ccccc} {}*20c0.7923 & 0.7924 & 0.7923 & 0.7922 & 0.7923 \end{array}\right].$$

Using the entropy-based weighting method of information indicators, to calculate the entropy weights of the characteristic values under different influencing factors, obtain the entropy weight function values for the positive and negative directions, sum the positive and negative values and take the average, normalize the resulting set, and calculate the entropy weight function value as:


$$w_{j}=\left[\begin{array}{ccccc} {}*20c0.2030 & 0.2054 & 0.1946 & 0.1920 & 0.2051 \end{array}\right].$$

Calculate the weighted membership matrix to obtain


$$X_{ij}=\left[\begin{array}{ccccc} {}*20c0.0131 & 0.0108 & 0.0283 & 0.0314 & 0.0107\\ 0.0199 & 0.0157 & 0.0329 & 0.0421 & 0.0167\\ 0.3993 & 0.3511 & 0.5985 & 0.6689 & 0.3597\\ 0.0003 & 0.0001 & 0.0000 & 0.0000 & 0.0001 \end{array}\right].$$

The maximum and minimum difference matrices can be obtained by this method:


$$X^{+}=\left[\begin{array}{ccccc} {}*20c0.3993 & 0.3511 & 0.5985 & 0.6689 & 0.3597 \end{array}\right],$$

$$X^{-}=\left[\begin{array}{ccccc} {}*20c0.0003 & 0.0001 & 0.0000 & 0.0000 & 0.0001 \end{array}\right].$$

The correlation coefficient $r_{ij}$ is a function related to $r_{ij}^{+}$ and $r_{ij}^{-}$. Therefore, when $r_{ij}^{+}$ and $r_{ij}^{-}$ fluctuate with the dataset, the correlation coefficient $r_{ij}$ also undergoes dynamic changes.

Finally, the comprehensive gray correlation coefficient $r_{ij}$ is calculated as:


$$r_{ij}=\left[\begin{array}{ccccc} {}*20c0.8215 & 0.8133 & 0.8449 & 0.8524 & 0.8152\\ 0.8176 & 0.8104 & 0.8427 & 0.8477 & 0.8116\\ 0.5596 & 0.5726 & 0.5137 & 0.5000 & 0.5703\\ 0.8287 & 0.8198 & 0.8580 & 0.8660 & 0.8215 \end{array}\right].$$

Under the combined influence of multiple factors, the gray correlation coefficient $r_{u}\left(I\;\right)$ of the wind turbine system status data is calculated, representing the uncertainty of each indicator under the influence of multiple factors.


$$r_{u}\left(I\;\right)=\left[\begin{array}{ccccc} {}*20c0.1989 & 0.1978 & 0.2022 & 0.2031 & 0.1980 \end{array}\right]$$

The value matrix of the evidence fusion function under the influence of multiple factors is calculated as:


$$m_{j}\left(i\right)=\left[\begin{array}{ccccc} {}*20c0.0105 & 0.0087 & 0.0225 & 0.0250 & 0.0086\\ 0.0160 & 0.0126 & 0.0263 & 0.0335 & 0.0134\\ 0.3199 & 0.2817 & 0.4775 & 0.5331 & 0.2885\\ 0.0003 & 0.0001 & 0.000 & 0.0000 & 0.0001 \end{array}\right]$$

**Fig 3:**
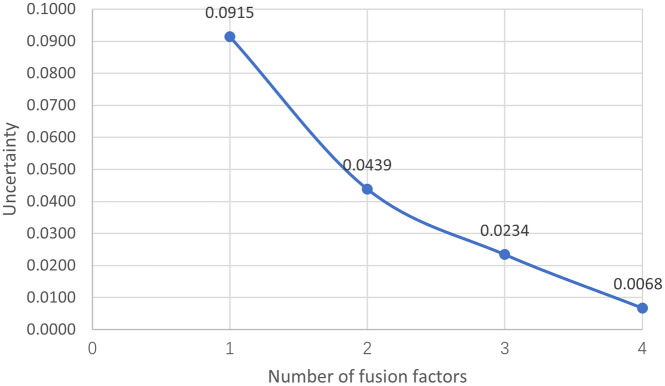
Trend curve of uncertainty changes.

The gearbox system status can be determined through fusion analysis of the uncertainty matrix of the evidence function. The improved entropy-weighted GRA and D-S theory fusion algorithm was used to calculate system state reliability, yielding the credibility function matrix $Bel\left(A\right)=\left[\begin{array}{ccccc} {}*20c0.3446 & 0.3030 & 0.5263 & 0.5916 & 0.3106 \end{array}\right]$. The uncertainty probability matrix under multi-factor fusion is $\left[\begin{array}{cccc} {}*20c0.0915 & 0.0439 & 0.0234 & 0.0068 \end{array}\right]$. The final result that during the process of transitioning from single-factor judgment to multi-factor fusion, the uncertainty probability of the function decreased from 9.15% to 0.68%. To a certain extent, the proposed algorithm has improved the accuracy of assessing the reliability of the system state. The specific uncertainty trend curve is shown in Fig 3. The reliability assessment of gearboxes and wind turbines can be positively supported by the use of parameter features in calculations, as indicated.

Due to the uncertainty of the reliability of each indicator due to the influence of multiple factors, feature fusion analysis is carried out. The new fusion algorithm evaluates the reliability of the gearbox status of the wind turbine system. In the *F*_*2*_ and *F*_*3*_ states, points less than 1 are rounded up using the ROUNDUP (number, 0) function. Table 3 displays the final judgment results.

**Table 3:** Judgment results of the reliability of the gearbox status of the wind turbine system.

Analysis data Actual data	*F*_*0*_	*F*_*1*_	*F*_*2*_	*F*_*3*_
*F*_*0*_	130	4	0	0
*F*_*1*_	0	60	0	0
*F*_*2*_	0	1	1	0
*F*_*3*_	0	1	0	1

As shown in Table 3, after analysis using the improved entropy-weighted GRA and D-S theory algorithm based on interval feature values, there are 130 samples in the normal state interval, 68 samples in the latent state interval, 1 sample in the risk state interval, and 1 sample in the alarm state interval. The overall identification accuracy is 97%, which is 4.5% more than the traditional algorithm. The comparison between the initial algorithm, the improved algorithm, and the actual values for the determination of gearbox status of the wind turbine system is shown in Table 4.

**Table 4:** Comparison between the initial algorithm, the improved algorithm, and the actual values for the gearbox status determination of the wind turbine system.

Status Data	*F*_*0*_	*F*_*1*_	*F*_*2*_	*F*_*3*_
*Original*	145	53	1	1
*Improved*	130	68	1	1
*Actual*	134	62	2	1

**Fig 4:**
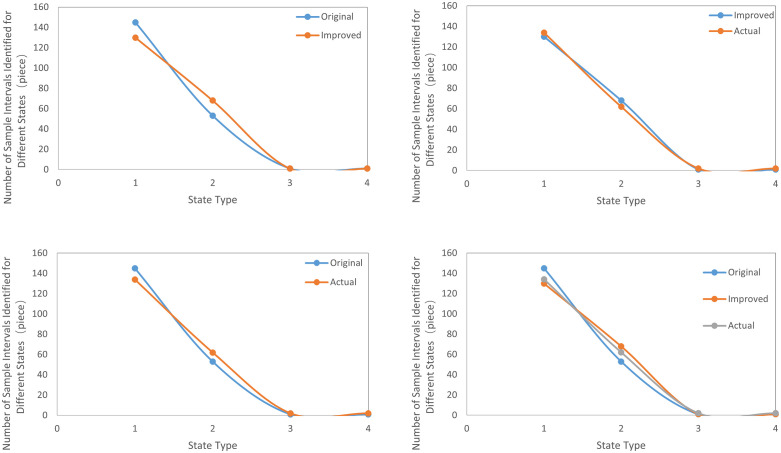
Fitting analysis curves. **(a)** Fitting of the original algorithm results and the improved algorithm. **(b)**Fitting of the improved algorithm results and the actual values. **(c)** Fitting of the original algorithm results and the actual values. **(d)** Fitting of the original algorithm, improved algorithm results, and actual values.

Fig 4 displays the fitting curves for the initial algorithm, the improved algorithm, and the actual values.

Through calculations of the confidence function matrix and uncertainty probability matrix, and by comparing the gearbox status assessments of the wind turbine system under the initial algorithm and the improved algorithm with actual values, it is evident that introducing adaptive entropy-weighted grey correlation enhances the algorithm’s sensitivity compared to the original function sensitivity, thereby effectively improving evaluation accuracy.

## Conclusions

Nonlinear systems are characterized by complex structures and operational environments, with diverse tasks. System state information can be uncertain due to ambiguity, conflict, gray areas, and randomness. Low accuracy in identifying system state anomalies and challenges for system state reliability analysis impact the efficient operation of the system.

Based on previous research, this paper combines GRA, entropy, and D-S theory, considering changes in factor weights in uncertain environments. It employs a multi-factor weight adaptive dynamic adjustment model to construct an entropy-weighted gray correlation state reliability analysis algorithm. This reduces the impact of information feature uncertainties on the reliability analysis of nonlinear system states and mitigates the analytical shortcomings of traditional D-S theory algorithms, thereby making the reliability assessment of nonlinear system states more accurate. Using a wind turbine system as an example to validate the algorithm’s effectiveness, it was found that the proposed algorithm can perform dynamic real-time analysis under continuously fluctuating gearbox interval parameters. The uncertainty probability decreased from 9.15% to 0.68% when switching from single-factor judgment to multi-factor fusion judgment. Compared to traditional D-S theory algorithms, the overall assessment accuracy improved by 4.5%, and the reliability probability analysis accuracy improved by 5.22%, better aligning with the actual system’s state probability distribution.

This method applies not only to the case study presented in this study but also to the reliability state analysis of other nonlinear systems influenced by multiple factors. Due to space constraints, this study leaves room for further research on the integration analysis of different combination rules. Future studies will build upon the preliminary research and expand the experimental investigations.

## Supporting information

S1 Appendix Data. (XLSX)
